# Mutations in *MC1R* Gene Determine Black Coat Color Phenotype in Chinese Sheep

**DOI:** 10.1155/2013/675382

**Published:** 2013-09-03

**Authors:** Guang-Li Yang, Dong-Li Fu, Xia Lang, Yu-Tao Wang, Shu-Ru Cheng, Su-Li Fang, Yu-Zhu Luo

**Affiliations:** ^1^Department of Life Sciences, Shangqiu Normal University, Shangqiu 476000, China; ^2^Gansu Province Key Laboratory of Herbivorous Animal Biotechnology, Gansu Agricultural University, Lanzhou 730070, China; ^3^Lanzhou Institute of Animal and Veterinary Pharmaceutics Sciences, Chinese Academy of Agricultural Sciences, Lanzhou 730050, China

## Abstract

The melanocortin receptor 1 (MC1R) plays a central role in regulation of animal coat color formation. In this study, we sequenced the complete coding region and parts of the 5′- and 3′-untranslated regions of the *MC1R* gene in Chinese sheep with completely white (Large-tailed Han sheep), black (Minxian Black-fur sheep), and brown coat colors (Kazakh Fat-Rumped sheep). The results showed five single nucleotide polymorphisms (SNPs): two non-synonymous mutations previously associated with coat color (c.218 T>A, p.73 Met>Lys. c.361 G>A, p.121 Asp>Asn) and three synonymous mutations (c.429 C>T, p.143 Tyr>Tyr; c.600 T>G, p.200 Leu>Leu. c.735 C>T, p.245 Ile>Ile). Meanwhile, all mutations were detected in Minxian Black-fur sheep. However, the two nonsynonymous mutation sites were not in all studied breeds (Large-tailed Han, Small-tailed Han, Gansu Alpine Merino, and China Merino breeds), all of which are in white coat. A single haplotype AATGT (haplotype3) was uniquely associated with black coat color in Minxian Black-fur breed (*P* = 9.72*E* − 72, chi-square test). The first and second A alleles in this haplotype 3 represent location at 218 and 361 positions, respectively. Our results suggest that the mutations of *MC1R* gene are associated with black coat color phenotype in Chinese sheep.

## 1. Introduction

Animal coloration is an ideal model for studying the genetic mechanisms that determine phenotype [1]. Coat color in domestic animals is one of the most strikingly variable and visible traits and has been widely used as a unique phenotype in the morphological selection for breed identification and attribution. In a large number of mammalian species, the coat color diversity is mainly determined by the relative amount of two basic melanins, eumelanin (black/brown), and pheomelanin (yellow/red), which are genetically controlled by the *Extension* (*E*) and *Agouti* (A) loci, respectively [2]. The *Agouti* locus encodes for the agouti signalling protein (ASIP) [3], a small paracrine signaling molecule that interacts with the product of the *Extension* locus. The *E* locus encodes the melanocortin receptor 1 (MC1R), which is a seven-transmembrane domains protein belonging to the G-protein coupled receptor present on the surface of the melanocyte membrane [4].

Functional mutations of the *MC1R* gene causing variation in coat colors have been described in domestic animals, such as cattle [5], pigs [6, 7], horses [8], goats [9], and sheep [10–13]. A notable example is the conserved role of the *MC1R* in mammalian pigmentation [14]. Studies of *MC1R* have provided valuable insights not only into the biology of pigmentation but also the evolution of domesticated animals [15, 16].

**Figure 1:**
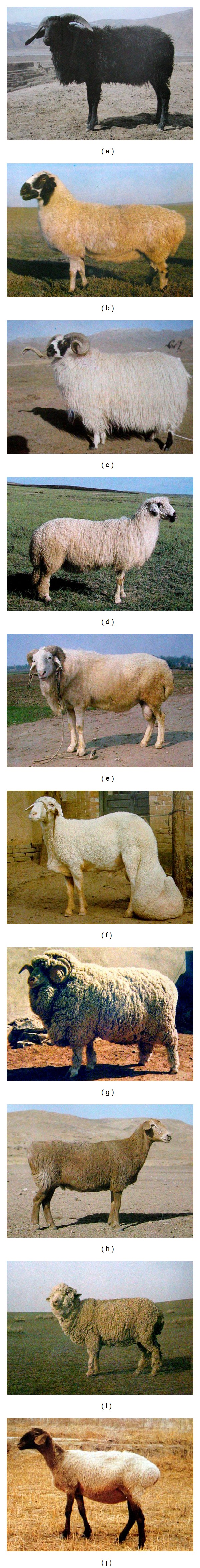
Illustration of sheep coat colors. (a) Minxian Black-fur sheep: black; (b) Mongolian sheep: white coat with black or brown face; (c) Tibetan sheep: white coat with black or brown face; (d) Tan sheep: white coat with black or brown face; (e) Small-tailed Han sheep: white; (f) Large-tailed Han: white; (g) China Merino: white; (h) Kazakh Fat-Rumped: Brown; (i) Gansu Alpine Merino: white; (j) Duolang sheep: white or gray coat with black or brown face.

China has more than 40 native sheep breeds [17]. During the long-term selective breeding, it has resulted in diverse coat color phenotypes in Chinese indigenous breeds, including black, white, and brown pigment types. There are three ecosystem sheep groups (Kazak, Tibetan, and Mongolian) in China as well as other local populations or breeds (Minxian Black-fur, Small-tailed Han, Large-tailed Han, Tan, Gansu Alpine Merino, China Merino, and Duolang) [17]. In most breeds, all animals share the same color pattern as breed character, such as Minxian Black-fur, Kazakh Fat-Rumped, and Mongolian sheep breeds (Ujimqin, Bayinbuluk, Wuranke, Sunite, and Hulun Buir) [17] (Figure 1). The Minxian Black-fur sheep is the predominant among Chinese indigenous breed that has uniform black coat [17].

In sheep, at least *ASIP*, *MC1R*, and *TYRP1* genes have been implicated in coat color [10–13, 18–21]. There are lots of papers describing the effect of *MC1R* gene in coat color trait [10–13]. *MC1R *gene is located on chromosome 14 (OAR14) in sheep [11] and has three main alleles (*E* ^+^, *E* ^*D*^, and *e*), which are defined by three mutations in the coding region and associated with variation in coat color [10–12]. However, so far there is no report regarding the *MC1R* gene and the potential association of its mutations with coat colors in Chinese indigenous sheep. Therefore, we characterized the *MC1R* gene by sequencing DNA pools comprising 30 sheep individuals belonging to three native breeds with different coat colors: Minxian Black-fur sheep (Black), Large-tailed Han (White), and Kazakh Fat-Rumped (Brown) (Figure 1) and subsequent analysis of mutations in 10 different Chinese sheep breeds. The purpose of this study was to investigate the variability in *MC1R* and their possible association with the coat color in Chinese sheep breeds.

## 2. Material and Methods

### 2.1. Animals

A total of 373 blood samples were collected from 10 Chinese sheep breeds representing a range of distinct coat colors (Figure 1). Breed name, sample size, coat color phenotype, and sampling location for each breed were shown in Table 1. Coat colors were determined by direct visual inspection. Genomic DNA was extracted from blood specimens by using the TIANamp blood DNA kit (Tianjin, Beijing, China).

### 2.2. SNPs Identification and Genotyping

SNPs were identified by sequencing amplicons of the whole coding domain sequences (CDS, 954 bp) and parts of the 5′- and 3′-untranslated regions (35 and 125 bp, resp.) of *MC1R* in both directions. Three DNA pools comprise thirty individuals with 10 individuals DNA (100 ng/*μ*L, 5 *μ*L for each individual) from each breed of Large-tailed Han sheep (White), Minxian Black-fur sheep (Black), and Kazakh Fat-Rumped sheep (Brown) and were used for identification mutation sites. Primers (MF: GAGAGCAAGCACCCTTTCCT, MR: GAGAGTCCTGTGATTCCCCT) for *MC1R* amplification and sequencing were designed with the program Primer 3 (http://fokker.wi.mit.edu/) based on the published coding region sequences in sheep (GenBank accession number: Y13965) and the complete sequences in bovine and goat which include 5′- and 3′-untranslated flanking regions (GenBank accession numbers: AF445641 and FM212940). 

All amplifications were performed on Eppendorf Mastercycler (Hamburg, Germany). The reaction was performed in a total of 25 *μ*L containing 50 ng DNA template (DNA pools), 100 *μ*M dNTPs, 10 pM of *MC1R* specific primers (MF and MR), and 2.5 U Taq polymerase (Bocai, Shanghai, China). After denaturation at 94°C for 3 min, 35 amplification cycles were performed comprising a denaturation step at 94°C for 30 s and an annealing step at 62°C for 30 s, an extension at 72°C for 45 s, followed by a last extension at 72°C for 10 min. The PCR products were separated and visualized by electrophoresis on 1.5% agarose gels ethidium bromide staining. PCR products were purified with the QIAquick PCR Purification Kit (Qiagen, Hilden, Germany). Sequences were analyzed using DNAStar software (DNAStar Inc., Madison, WI, USA) to identify polymorphisms. Identified highly informative SNPs were (minor allele frequencies >0.3) chosen for genotyping by sequencing in a larger sample of animals belonging to the 10 breeds. PCR amplification and SNPs genotyping were performed as described above.

### 2.3. Data Analysis

Deviations from Hardy-Weinberg equilibrium (HWE) between SNPs were tested by POPGENE 3.1 [22]. Haplotypes of the SNPs within *MC1R* gene were determined using the PHASE program v. 2.1 [23]. The association analyses between haplotypes and coat colors were performed using crosstabs with fisher exact test implemented in the procedure descriptive statistics with the SPSS version 16.0 software (SPSS Inc. Chicago, IL, USA).

## 3. Results

### 3.1. SNPs Identification and Genotyping

**Figure 2:**
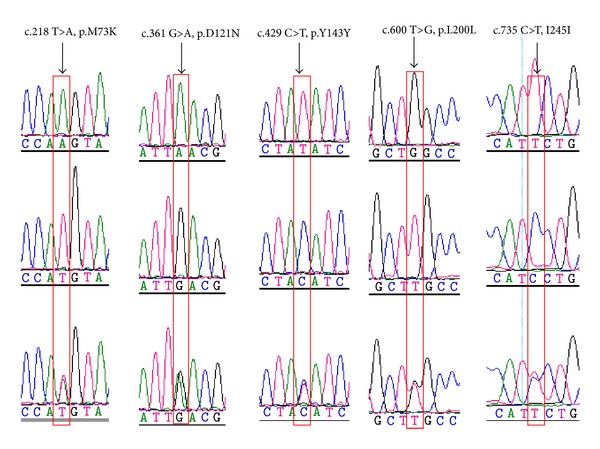
Identified SNPs and alignment of the MC1R protein regions around the deduced amino acid substitutions.

By analysing and comparing the obtained sequence electropherograms from DNA pools of 30 sheep individuals. The results showed that five single nucleotide polymorphisms (SNPs), two nonsynonymous mutations previously associated with coat color (c.218 T>A, p.73 Met>Lys. c.361 G>A, p.121 Asp>Asn) and three synonymous mutations (c.429 C>T, p.143 Tyr>Tyr; c.600 T>G, p.200 Leu>Leu. c.735 C>T, p.245 Ile>Ile), were identified in the CDS of *MC1R* gene (Figure 2) (GenBank accession number: KF198511). These polymorphisms were reported by Våge et al. [10] and Fontanesi et al. [12]. However, we did not find recessive allele *e* (c.199 C>T), which was reported by Fontanesi et al. [12].

These SNPs were further screened in a larger number of animals of 10 Chinese sheep breeds. Genotypes and allele frequencies were shown in Table 2. A chi-square test showed that 10 breeds were in Hardy-Weinberg equilibrium, while Kazakh Fat Rumped and Minxian Black-fur breed showed significant (*P* < 0.05) and very significant (*P* < 0.01) departures from Hardy-Weinberg equilibrium at *MC1R* c.218 T>A and *MC1R* c.361 G>A.

All mutation alleles (c.218A, c.361A, c.429T, c.600G, and c.735T) were detected in Minxian Black-fur sheep breed, and each mutation site has two genotypes. In particular, two nonsynonymous mutations (c.218 T>A, p.73 Met>Lys. c.361 G>A, p.121 Asp>Asn) determining the dominant black (*E* ^*D*^) allele [10], were not at all identified in Large-tailed Han, Small-tailed Han, Gansu Alpine Merino, and China Merino, all of which are in white coat color. The Kazakh Fat-Rumped and Mongolian populations have three genotypes for each nonsynonymous mutation loci. But two nonsynonymous mutations frequencies were very low or rare in Kazakh Fat-Rumped, Mongolian, and other three Chinese native sheep breeds (Tibetan, Tan, and Duolang). Three silent mutations in other sheep breeds, except for Minxian Black-fur and Kazakh Fat-Rumped breeds, have three genotypes. Interestingly, in this study, we found almost all mutation alleles in the Minxian Black-fur sheep breed at a rather high frequency (0.6630 and 0.8913). Three silent mutation alleles have also higher frequency (0.667) than two nonsynonymous mutation sites (0.333) in Kazakh Fat-Rumped breed. 

### 3.2. Haplotype

Table 3 reports individual diplotype types and haplotype frequencies among the investigated breeds. Three haplotypes (haplotype1 [TGCTC], haplotype2 [TGTGT], and haplotype3 [AATGT]) and six individual diplotype types (haplotype1/haplotype1, haplotype1/haplotype2,  haplotype2/haplotype2,  haplotype1/haplotype3, haplotype2/haplotype3, and haplotype3/haplotype3) were identified. The haplotype3 of the all individual mutations was observed only in the Minxian Black-fur sheep breed. 17 Minxian Black-fur sheep were homozygous for haplotype3/haplotype3, 29 Minxian Black-fur sheep were heterozygous for haplotype1/haplotype3 (10), and for haplotype2/haplotype3 (19). The white coat color breeds (Large-tailed Han, Small-tailed Han, Gansu Alpine Merino, and China Merino) were only found in three diplotype types (haplotype1/haplotype1, haplotype1/haplotype2, and haplotype2/haplotype2). The Mongolian has six diplotype types. Tan and Kazakh Fat-Rumped breeds have five diplotype types. Tibetan and Duolang have four similar diplotype types. Interestingly, we also observed that the haplotype3 frequency was the highest in Minxian Black-fur sheep population (0.6848). The haplotype3 was absent in four Chinese sheep breeds with white coat (Large-tailed Han, Small-tailed Han, Gansu Alpine Merino, and China Merino) and was very low (0.0119–0.333) in other five Chinese indigenous breeds (Tibetan, Mongolian, Tan, Kazakh Fat-Rumped, and Duolang).

### 3.3. Association Analysis

Among a total of 373 Chinese sheep individuals, 46 were black coat color phenotype (Minxian Black-fur) and 18 were classified as brown (Kazakh Fat-Rumped). The genotyping and haplotyping data (Tables 2 and 3) clearly indicated that polymorphisms in the *MC1R* gene affect coat color in Minxian Black-fur. First of all, all animals with a mutation sites haplotype3 (46) showed uniform apparent black coat color, and almost all animals without a mutation sites haplotype3 (143) were completely white coat color (Large-tailed Han, Small-tailed Han, Gansu Alpine Merino, and China Merino). Secondly, the association analyses between haplotypes and coat colors are also showing that all the mutation alleles of haplotype3 were highly significantly associated with Minxian Black-fur coat color (*P* = 9.72*E* − 72). But only a few animals did not follow the above rules: twenty-one of 184 (13/166 white coat color with black or brown patches in the head and 8/18 brown animals) carried out haplotype3. The alleles of haplotype3 have not been associated with white coat color with black or brown patches in the head (Tibetan, Mongolian, Tan, and Duolang) and brown coat color (Kazakh Fat-Rumped) animals.

## 4. Discussion

Classical genetic studies had proved two alleles (*E* ^*D*^ and *E* ^+^) at the *Extension* locus affecting sheep coat color phenotypes [2, 24]. Subsequently, Våge et al. [10] characterized two missense mutations (p.M73 K and p.D121N) determining the dominant black (*E* ^*D*^) allele in the Norwegian Dala breed. The presence of two mutations was also observed in other sheep breeds: Corriedale, Damara, Black Merino, Black Castellana, and Karakul [11, 19]. The allele *E* ^*D*^ was directly involved in affecting sheep pigmentation at the molecular level and causes the dominant black coat color.

The recessive *e* allele of the *Extension* locus has also been clearly documented in sheep. One SNP (c.199 C>T) caused a predicted amino acid substitution (p.R67C) in a highly conserved position of the first intracellular loop of the MC1R protein [12]. The same substitution causes recessive pheomelanism in other species [7, 25]. Therefore, they propose that the p.67C allele represents the recessive *e* allele at the sheep *Extension* series that was not completely recognized in sheep by classical genetic studies. This polymorphism was analysed in Italian sheep breeds or populations. Confirming the effect of this novel allele on coat color will lead to new perspectives. 

Chinese sheep breeds have more variations on coat color among and, in some cases, within breeds. Therefore, five SNPs were also identified in Chinese sheep breeds in the *MC1R* gene by direct sequencing (Figure 2). The recessive allele *e* (c.199 C>T), which has been linked to the control coat color in sheep, was not detected in the Chinese sheep. Two of five polymorphisms (c.218 T>A and c.361 G>A) were deduced as nonsynonymous substitutions causing a p.M73K and the p.D121N amino acid change, respectively. In the sheep, two amino acid (p.M73K and p.D121N) changes resided in the extracellular second transmembrane region (p.M73K) and in the third transmembrane domain (p.D121N) [10]. Both mutations in sheep have been associated with coat color variation. Additionally, both mutations could explain the dominant black coat color in sheep [10, 11, 19].

Five SNPs were genotyped in 10 Chinese sheep breeds with different coat color phenotypes. All mutations were detected in Minxian Black-fur sheep breed, and nonsynonymous mutation sites were not at all identified in white coat coloration breeds (Table 2). This finding demonstrated that five mutations were completely associated with the black coat color in Minxian Black-fur sheep population. Meanwhile, three haplotypes (haplotype1, haplotype2, and haplotype3) were defined by the mutations SNPs in the *MC1R* gene. It was interesting that haplotype3 was almost fixed in the Minxian Black-fur sheep breed (two missense mutations causing the *E* ^*D*^ allele were inserted in a haplotype3). Other four completely white sheep breeds had not carried the haplotype3 (Table 3). Furthermore, association analysis also indicated that the alleles of haplotype3 were significantly associated with the black coat color (*P* = 9.72*E* − 72, Chi-square test). Therefore, the alleles of haplotype3 might be a possible result that can interpret black coat color mechanisms in the Minxian Black-fur sheep breed that shaped the genetic pool of this sheep breed.

In Kazakh Fat-Rumped, two nonsynonymous mutation sites have three genotypes, and three silent mutations results were in accordance with Minxian Black-fur sheep breed (Table 2). Moreover, this breed has five diplotype types (Table 3). Thus, it is worthwhile to caution that the brown phenotype in Kazakh Fat-Rumped breed seems not to be caused by the identified *MC1R* mutations. However, Gratten et al. [20] report the identification of the *TYRP1* gene and causal mutation underlying coat color variation in a free-living population of Soay sheep. They identified a nonsynonymous substitution in exon IV that was perfectly associated with coat color. This polymorphism is predicted to cause the loss of a cysteine residue that is highly evolutionarily conserved and likely to be of functional significance. They eliminated the possibility that this association is due to the presence of strong linkage disequilibrium with an unknown regulatory mutation by demonstrating that there is no difference in relative TYRP1 expression between color morphs. Analysis of this putative causal mutation in a complex pedigree of more than 500 sheep revealed almost perfect cosegregation with coat color and very tight linkage between coat color and TYRP1.

In addition, according to the phenotype observed in Chinese-Tibetan having the same brown coat color [26], Ren et al. [27] performed a genome-wide association study (GWAS) on Tibetan and Kele pigs and found that brown colors in Chinese breeds are controlled by a single locus on pig chromosome 1. Then, by using a haplotype-sharing analysis, they refined the critical region to a 1.5 Mb interval that encompasses only one pigmentation gene: *TYRP1*. Lastly, mutation screens of sequence variants in the coding region of *TYRP1* revealed a strong candidate causative mutation (c.1484-1489del). The protein-altering deletion showed complete association with the brown coloration across Chinese-Tibetan, Kele, and Dahe breeds by occurring exclusively in brown pigs and lacking in all nonbrown-coated pigs from 27 different breeds. The findings provide the compelling evidence that brown colors in Chinese indigenous pigs are caused by the same ancestral mutation in TYRP1. Moreover, Beraldi et al. [28] have shown an effect of dilution of pigmentation in Soay sheep that maps to chromosome 2, in a region where the candidate gene for brown coat color, *TYRP1*, is located. Therefore, we can rule out the possibility of *MC1R* mutations determining the brown coat color phenotype. The brown coat color phenotype in Kazakh Fat-Rumped sheep may be caused by *TYRP1* gene mutations that need to be further investigated.

Tibetan, Duolang, Tan, and Mongolian breeds usually include completely white coat animals together with black or brown patches in the head (around the eyes and/or in the ears or cheeks) (Figure 1). According to results from genotype and haplotype, the same substitution and haplotype (haplotype frequencies) were present in Tibetan, Duolang, Tan, and Mongolian breeds. However, there was no complete association between the presence of black or brown spots in the face and the presence of the AATGT alleles or haplotype3. Fontanesi et al. [9] reported missense and nonsense mutations in *MC1R* gene of different goat breeds. According to the results obtained that *MC1R* mutations may determine eumelanic and pheomelanic phenotypes, however, they are probably not the only factors. In particular, the surprising not complete association of the nonsense mutation (p.Q225X) with red coat colour raises a few hypotheses on the determination of pheomelanic phenotypes in goats that should be further investigated. Sponenberg et al. [24] showed that the wild allele at the Spotting locus allows full extension of pigmentation with no white spotting. The spotting of the recessive allele usually involves the distal legs and top of head before other areas and tends to result in reasonably recognizable patterns of spotting. Adalsteinsson [29] also suggested that the variation in the spotted (S^S^) effect can be explained by the action of modifiers, and white head spot occurs in animal heterozygous for white markings by incomplete dominance of the dominant allele (S^+^) for full pigmentation. Hence, these (Tibetan, Duolang, Tan, and Mongolian) breeds were probably due to incomplete fixation of different alleles at the spotting locus. The spotting locus or other loci with similar phenotypic effects might act through inhibition or disregulation of melanocyte migration from the neural crest at the embryonic level. This complicates the interpretation of the results as a complete characterization of the spotting locus in sheep is lacking. Therefore, when spots are present it could be possible to evaluate if different mutations are associated with the presence of eumelanic or pheomelanic colors.

Norris and Whan [18] characterized the sheep *ASIP* gene showing that a 190 kb tandem duplication encompassing this gene, the AHCY coding region (CDS), and the ITCH promoter region should be the cause of the white coat colour of dominant white and tan (A^Wt^) Agouti sheep. In addition, a not yet characterized regulatory mutation as well as a deletion of 5 bp in exon 2 and a missense mutation in exon 4 was identified as the causes of the black recessive nonagouti (A^a^) allele [18, 19, 21]. Analysis of the *ASIP* gene was also performed in the same Chinese sheep breeds by Fu et al. [30] (in press). The results showed that two deletion mutations and three SNPs were identified: a 9 bp deletion (c.10-18del) and 5-bp deletion (c.100-105del), both of which were located in exon 2, and three SNPs (g.672 G>A, g.1580 G>A and g.1617 G>A) were located in intron 2. Two deletion mutations were presented in 10 Chinese sheep breeds. Moreover, only two sheep have the D_5_D_5_ genotype, one in Minxian Black-fur sheep and one in Duolang sheep, and no homozygosis D_9_D_9_ was found in all sheep that we detected. The genotype results suggested that these mutations are not associated or not completely associated with coat color in the investigated sheep breeds. The above results indicated that the variation in the protein coding region of *ASIP* did not explain the coat colour phenotypes variation of Chinese indigenous sheep breeds. These investigated results are also proved evidence that the black coat color phenotype in Chinese sheep was caused by the *MC1R* gene mutations.

## 5. Conclusion

The present study results further confirm that the *MC1R* gene is an important candidate gene because its mutations are associated with black color phenotype in Chinese indigenous sheep breed. In addition, we can rule out the mutations of *MC1R* determining the brown coat color phenotype.

## Figures and Tables

**Table 1:** Sample collection: breed name, sample size, coat color phenotype, and sampling location.

Breed	Number	Coat color phenotype	Sampling location
Minxian Black-fur	46	Black	Gansu, Min county
Tibetan	42	White, with black or brown face	Qinghai, Hainan county
Large-tailed Han	48	White	Henan, Jia county
Small-tailed Han	34	White	Shandong, Heze county
Mongolian	51	White, with black or brown face	Mongolian
Tan	45	White, with black or brown face	Gansu, Jintai county
Kazakh Fat-Rumped	18	Brown	Xinjiang, Akesu
Gansu Alpine Merino	34	White	Gansu, Sunan county
China Merino	27	White	Xinjiang, Yili state
Duolang	28	White or gray, with black or brown face	Xinjiang, Maigaiti county

**Table 2:** Genotype and allele frequencies of the 5 SNPs in *MC1R* in Chinese sheep breeds.

Breed	*MC1R1* c.218 T>A					*MC1R1* c.361 G>A					*MC1R1* c.429 C>T					*MC1R1* c.600 T>G					*MC1R1* c.735 C>T				
	Genotype			Allele frequency		Genotype			Allele frequency		Genotype			Allele frequency		Genotype			Allele frequency		Genotype			Allele frequency	
	AA	AT	TT	A	T	AA	AG	GG	A	G	TT	CT	CC	T	C	GG	GT	TT	G	T	TT	TC	CC	T	C
Minxian Black-fur	17	29	0	0.6630	0.3370	17	29	0	0.6630	0.3370	36	10	0	0.8913	0.1087	36	10	0	0.8913	0.1087	36	10	0	0.8913	0.1087
Tibetan	0	1	41	0.0119	0.9881	0	1	41	0.0119	0.9881	17	20	5	0.6429	0.3571	17	20	5	0.6429	0.3571	17	20	5	0.6429	0.3571
Large-tailed Han	0	0	48	0	1	0	0	48	0	1	2	14	32	0.1875	0.8125	2	14	32	0.1875	0.8125	2	14	32	0.1875	0.8125
Small-tailed Han	0	0	34	0	1	0	0	34	0	1	10	16	8	0.5294	0.4706	10	16	8	0.5294	0.4706	10	16	8	0.5294	0.4706
Mongolian	1	8	42	0.0980	0.9020	1	8	42	0.0980	0.9020	9	28	14	0.4510	0.5490	9	28	14	0.4510	0.5490	9	28	14	0.4510	0.5490
Tan	0	2	43	0.0222	0.9778	0	2	43	0.0222	0.9778	8	20	17	0.4000	0.6000	8	20	17	0.4000	0.6000	8	20	17	0.4000	0.6000
Kazakh Fat-Rumped	2	8	8	0.3333	0.6667	2	8	8	0.3333	0.6667	6	12	0	0.6667	0.3333	6	12	0	0.6667	0.3333	6	12	0	0.6667	0.3333
Gansu Alpine Merino	0	0	34	0	1	0	0	34	0	1	6	17	11	0.4265	0.5735	6	17	11	0.4265	0.5735	6	17	11	0.4265	0.5735
China Merino	0	0	27	0	1	0	0	27	0	1	1	16	10	0.3333	0.6667	1	16	10	0.3333	0.6667	1	16	10	0.3333	0.6667
Duolang	0	1	27	0.0179	0.9821	0	1	27	0.0179	0.9821	6	14	8	0.4643	0.5357	6	14	8	0.4643	0.5357	6	14	8	0.4643	0.5357

**Table 3:** Haplotype and haplotype frequencies at *MC1R* in 10 Chinese sheep breeds.

Breed	Haplotype						Haplotype frequency		
	1/1	1/2	2/2	1/3	2/3	3/3	Haplotype1 [TGCTC]	Haplotype2 [TGTGT]	Haplotype3 [AATGT]
Minxian Black-fur	0	0	0	**10**	**19**	**17**	0.1087	0.2065	0.6848
Tibetan	5	18	18	0	1	0	0.3571	0.6310	0.0119
Large-tailed Han	**32**	**14**	**2**	0	0	0	0.8125	0.1875	0.0000
Small-tailed Han	**8**	**16**	**10**	0	0	0	0.4706	0.5294	0.0000
Mongolian	14	22	6	6	2	1	0.5490	0.3529	0.0981
Tan	17	19	7	1	1	0	0.6000	0.3778	0.2222
Kazakh Fat-Rumped	0	7	1	5	3	2	0.3333	0.3333	0.3333
Gansu Alpine Merino	**11**	**18**	**5**	0	0	0	0.5735	0.4265	0.0000
China Merino	**9**	**16**	**2**	0	0	0	0.6667	0.3333	0.0000
Duolang	8	14	5	0	1	0	0.5357	0.4464	0.0179

Notes: Haplotypes are indicated following the SNP positions in the *MC1R* gene: c.218 T>A, c.361 G>A, c.429 C>T, c.600 T>G, and c.735 C>T.
